# Quantifying polymorphism and divergence from epigenetic data: a framework for inferring the action of selection

**DOI:** 10.3389/fgene.2015.00190

**Published:** 2015-05-28

**Authors:** Shivani Mahajan, Jessica Crisci, Alex Wong, Schahram Akbarian, Matthieu Foll, Jeffrey D. Jensen

**Affiliations:** ^1^School of Life Sciences, École Polytechnique Fédérale de LausanneLausanne, Switzerland; ^2^Swiss Institute of BioinformaticsLausanne, Switzerland; ^3^Department of Molecular and Computational Biology, University of Southern CaliforniaLos Angeles, CA, USA; ^4^Department of Biology, Carleton UniversityOttawa, ON, Canada; ^5^Brudnick Neuropsychiatric Research Institute, University of Massachusetts Medical SchoolWorcester, MA, USA; ^6^Departments of Psychiatry and Neuroscience, Friedman Brain Institute, Icahn School of Medicine at Mount SinaiNew York, NY, USA

**Keywords:** adaptation, epigenetics, epi-F_ST_, ANEVA

## Abstract

Epigenetic modifications are alterations that regulate gene expression without modifying the underlying DNA sequence. DNA methylation and histone modifications, for example, are capable of spatial and temporal regulation of expression—with several studies demonstrating that these epigenetic marks are heritable. Thus, like DNA sequence, epigenetic marks are capable of storing information and passing it from one generation to the next. Because the epigenome is dynamic and epigenetic modifications can respond to external environmental stimuli, such changes may play an important role in adaptive evolution. While recent studies provide strong evidence for species-specific signatures of epigenetic marks, little is known about the mechanisms by which such modifications evolve. In order to address this question, we analyze the genome wide distribution of an epigenetic histone mark (H3K4me3) in prefrontal cortex neurons of humans, chimps and rhesus macaques. We develop a novel statistical framework to quantify within- and between-species variation in histone methylation patterns, using an ANOVA-based method and defining an F_ST_ -like measure for epigenetics (termed epi- F_ST_), in order to develop a deeper understanding of the evolutionary pressures acting on epigenetic variation. Results demonstrate that genes with high epigenetic F_ST_ values are indeed significantly overrepresented among genes that are differentially expressed between species, and we observe only a weak correlation with SNP density.

## Introduction

Elucidating the relationship between genotypes and phenotypes remains an important challenge. One important question that remains in order to make further progress on this front is the ability to quantify why cells in different tissues, despite having the same DNA sequence, express different genes and maintain their distinct cellular identities. One of the key processes used to explain this paradox is epigenetics. Epigenetic modifications (including covalent modifications of the DNA and histone proteins, as well as RNA interference) regulate and alter the expression of genes without altering the underlying DNA sequence (Bird, 2007; Goldberg et al., 2007). Thus, these “epi-allele” modifications may provide an important source of variation within a population on which selection may act upon an environmental change.

One particularly interesting aspect of these modifications is their ability to escape reprogramming during gametogenesis and embryogenesis, and thus be propagated from parents to offspring (Jablonka and Lamb, 1998; Daxinger and Whitlelaw, 2010). In their review of transgenerational epigenetics in several taxa, Jablonka and Raz (2009) suggested that epigenetic inheritance may be ubiquitous. Most studies to date have focused on DNA methylation—the mode of transmission that is perhaps best understood. One widely studied example is the phenomenon of genomic imprinting, where the expression of a gene depends on the parent from which the gene was derived (Bell and Felsenfeld, 2000; Hark et al., 2000). For example, studies in mice have shown that genetically identical parents having different methylated states at Agouti can produce offspring with different coat colors (Morgan et al., 1999). Another recent study demonstrated that when mice were taught to fear an odor, this response was transmissible for up to two generations and was linked to changes in the DNA methylation status of a gene in the germline (Dias and Ressler, 2014).

Thus, although there is accumulating evidence in favor of epigenetic modifications being transmitted from parents to offspring, little is known about their evolutionary history or the selective forces acting upon them. Some studies have worked to incorporate epigenetic effects into models of natural selection and phenotypic evolution (Cowley and Atchley, 1992; Geoghegan and Spencer, 2012; Bonduriansky and Day, 2013), and a recent model also incorporates the effects of environmental change (Furrow and Feldman, 2014). Models have also been proposed to study trans-generational epigenetic inheritance and its effects on disease risk (Slatkin, 2009). All of these studies indicate that including epigenetic inheritance into traditional population genetics models has important consequences for adaptive evolution (e.g., faster rates of phenotypic evolution).

Jablonka and Raz (2009) discussed several ways in which heritable epigenetic markers could bring about evolutionary change, two of which are particularly interesting for the purposes here: selection acting directly on epigenetic variation, and epigenetic modifications guiding the selection of genetic variants. However, little work has been done to quantify the extent of natural variation in epigenetic markers. One recent study involving three human populations revealed population-specific differences in DNA methylation at certain CpG sites which were not correlated with sequence variation (Heyn et al., 2013). Human-specific selection signatures of H3K4me3 near the transcription start sites (TSSs) of prefrontal cortex neurons have also been described recently (Shulha et al., 2012).

As a necessary first step toward disentangling the effects of selection on epigenetic states and selection on underlying DNA variation, we present a novel statistical framework to quantify the within- and between-species variation observed using an ANOVA-based method analogous to the classical population genetic F_ST_ statistic (Excoffier et al., 1992). We examine the patterns of H3K4me3 enrichment in the prefrontal cortex neurons of humans, chimps and rhesus macaques. We identify the most epigenetically divergent genes between humans and chimps and study how this divergence correlates with differences in gene expression patterns observed between these species. This approach is akin to genome-wide scans for nucleotide and amino acid divergence, and can enable us to address questions including how frequently selection acts on epigenetic variation and whether this selection is indeed independent of DNA sequence variation. The framework presented here can easily be extended to include other epigenetic marks, in order to broaden our view of how the epigenetic landscape evolves, and whether these changes are species-specific and of potential adaptive importance.

## Methods

### Data

We use the ChIP-seq dataset from Shulha et al. (2012), which identified a total of 34,683 H3K4me3 histone methylation peaks in prefrontal cortex neurons of 11 humans (including adults and children), 4 chimps, and 3 rhesus macaques. The peaks were called by aligning Chip-seq reads from all three species to the human genome (assembly version hg19). Each peak represents a region of the genome that is enriched for H3K4me3, with the peak density corresponding to the strength of the enrichment. RNA-seq data (paired end 46 and 50 bp) from white and gray brain matter for 5 and 4 human individuals, respectively, were used from the above dataset. Single Nucleotide Polymorphism (SNP) data for the three species were downloaded from (http://www.ncbi.nlm.nih.gov/SNP/). TSS data for humans (hg19) was downloaded from the Ensembl database (http://www.ensembl.org/). Only TSSs for protein coding genes were retained for further analysis. Liftover (http://hgdownload.cse.ucsc.edu/admin/exe/) was used to obtain the TSS coordinates for chimps (panTro2) and macaques (rheMac2) from the human TSS data. Data for the gene expression differences between humans, chimps, and macaques was taken from Cain et al. (2011).

### Principal component analysis

Principal component analysis has been widely used in population genetics to detect population structure and to study genetic variation geographically, and can be useful in correcting for stratification when performing genome-wide association studies (Reich et al., 2008). The R package ade4 (Chessel et al., 2004) was used to perform principal component analysis (PCA) to identify groupings and/or clustering among the individuals of the three species based on the normalized peak density values of H3K4me3 density. We used an unscaled and centered PCA to avoid masking species-specific variation; therefore, the sum of the eigenvalues equals the total variance and the PCA corresponds to an eigenanalysis of the covariance matrix. We first performed a genome-wide PCA taking into account all histone peaks in chromosomes 1–22. Second, we used a sliding window approach with a window size of 1 Mb, sliding 100 kb. In each window, we calculated the pairwise Euclidean distances between the centers of the ellipses of dispersion of each species.

### ANEVA (analysis of epigenetic variance)

To quantify epigenetic variation within and between species we propose an ANOVA framework similar to AMOVA for genetic data (Excoffier et al., 1992). AMOVA can be used for a variety of molecular data to make inferences on population differentiation. The model used in the current study is as follows: let the normalized peak density *Y_ij_* be written as:

(1) $$Y_{ij}=\mu +\alpha_{i}+\varepsilon_{ij}$$

where μ is the expected mean peak density, α*_i_* is the species effect with the corresponding variance component σ^2^_α_ and ε_*ij*_ the individual or within-species effect with the corresponding variance component σ^2^_ε_.

All effects are assumed to be random and additive and the total sum of squares (SS) can be partitioned into the between-species and within-species components (Table 1):

(2) $$SS_{total}=SS_{between}+SS_{within}$$

**Table 1:** **Application of the ANOVA method for H3K4me3 peaks in humans and chimps**.

**Source of variation**	**D.F.**	**Sum of squares (SS)**	**Mean square (MS)**	**Expected MS**
Between species	1	Σ*_i_*(*y_i_* − *y*)^2^*n_i_*	SS(between)/1	σ^2^ + *n*_0_σ^2^_α_
Within species	13	Σ*_i_* Σ_*j*_ (*y_ij_* − *y_i_*)^2^	SS(within)/13	σ^2^
Total	14	SS(between) + SS(within)		

*Where n_0_ = [T − (Σ_i_n^2^_i_/T)]/(N − 1), with T = 15 (the total number of human and chimp individuals) and N = 2 (the number of species). Since we have an unbalanced ANOVA, with each species group having a different number of individuals, we calculate the effective sample size n_0_. Here n_0_ = 6*.

We define an F_ST_ -like measure for epigenetics based on analogy with AMOVA (Excoffier et al., 1992):

(3) $$epi-F_{st}\;=\;\frac{\sigma_{\alpha }^{2}\;+\;\sigma_{\varepsilon }^{2}}{\sigma^{2}}$$

From the ANOVA table (Table 1) we can calculate the natural estimates, written as *S*^2^ and *S*^2^_α_ using:

(4) $$S^{2}=\frac{SS_{within}}{dof_{within}}\;=\;\frac{\sum_{i}\sum_{j}{\left(y_{ij}-y_{i}\right)}^{2}}{dof_{within}}$$

(5) $$S_{\alpha }{}^{2}\;=\;\frac{SS_{between}-S^{2}}{n_{0}}\;=\;\frac{\sum_{i}{\left(y_{i}-\bar{y}\right)}^{2}n_{i}-S^{2}}{n_{0}}$$

where *y_i_*. represents the mean for the i^th^ species, *n_i_* is the number of individuals of the i^th^ species, *y* represents the overall grand mean, and *n*_0_ represents the effective sample size.

In this manner we can calculate an epi- F_ST_ statistic for each peak. As proposed by Excoffier et al. (1992), we used 10000 random permutations of individuals between species to assess significance of epi- F_ST_. Note that like in the AMOVA framework, our epi- F_ST_ values can be negative in some cases. These cases occur for very low values of *SS_within_*, and in our dataset if *SS_within_* = 0, we have the minimum possible epi- F_ST_ = −0.2 as *n*_0_ = 6 (see Table 1). As *n*_0_ → ∞, the minimum possible epi-F_ST_ → 0. We also calculated one epigenetic F_ST_ for each gene by averaging the epi- F_ST_ of all H3K4me3 peaks that lie within that gene or 50 kb upstream or downstream from the start and end of the gene, respectively.

### Identifying genes in regions that are variable within a species

The *SS_within_* calculated above can be further decomposed as:

(6) $$SS_{within}=\;SS_{within\_humans}\;+\;SS_{within\_chimps}$$

and for each peak we calculated the variance within humans and within chimps using:

(7) $$Var_{within\_humans}\;=\;\frac{SS_{within\_humans}}{n_{h}-1}$$

(8) $$Var_{within\_chimps}\;=\;\frac{SS_{within\_chimps}}{n_{c}-1}$$

where *n_h_* and *n_c_* are the number of human and chimp individuals, respectively.

Similar to the calculation method for the epi- F_ST_ for each gene, we calculated the variance in H3K4me3 enrichment for each gene by averaging the variance for all peaks lying within a gene or 50 kb upstream and downstream from its start and end, respectively.

### Expression analysis

Quality control of the paired-end illumina RNA-seq reads was performed using FastQC (http://www.bioinformatics.babraham.ac.uk/projects/fastqc/). Trimmed illumina paired-end RNA-seq reads were mapped to the human genome hg19 using tophat2 (Kim et al., 2013). Then, differential expression analysis was done using cuffdiff (Trapnell et al., 2010). For both mapping and differential expression analysis, the reference annotation was downloaded from (https://ccb.jhu.edu/software/tophat/index.shtml).

## Results and discussion

### Enrichment of H3K4me3 peaks near transcription start sites

To characterize the conservation of peaks near TSSs, we downloaded data for human (hg19) TSSs from Ensembl biomart, and limited our analysis to protein-coding genes only. Liftover was used to obtain the coordinates of the TSSs in chimps and macaques. As the peaks were obtained by mapping the ChIP seq reads to the human genome, we used Liftover to obtain the peak coordinates in the chimp and macaque genomes. Previous studies have shown that there is a strong overlap in H3K4me3 associated regions between these species (as much as ~70% between humans and chimpanzees and ~64% between humans and macaques; Cain et al., 2011). We found that ~46, ~37, and ~21% of the peaks were located within 2 Kb upstream or downstream of TSSs in humans, chimps, and macaques, respectively, when considering only unique TSSs (a single TSS per gene that corresponds to the longest transcript for that gene). When all known TSSs for a gene were considered, ~54, ~43, and ~22% of peaks in humans, chimps, and macaques, respectively, were found to be conserved near TSSs (2 kb upstream or downstream). Similarly, Cain et al. (2011) reported that 61.2 ± 1.5% were conserved near the TSSs in these three species. This difference could be due to the fact that we used only protein coding genes in our analysis, or owing to differences in H3K4me3 patterns between lymphoblastoid cell lines (LCL) used in their analysis and PFC neurons used here.

### Quantifying variation

#### PCA

**Figure 1:**
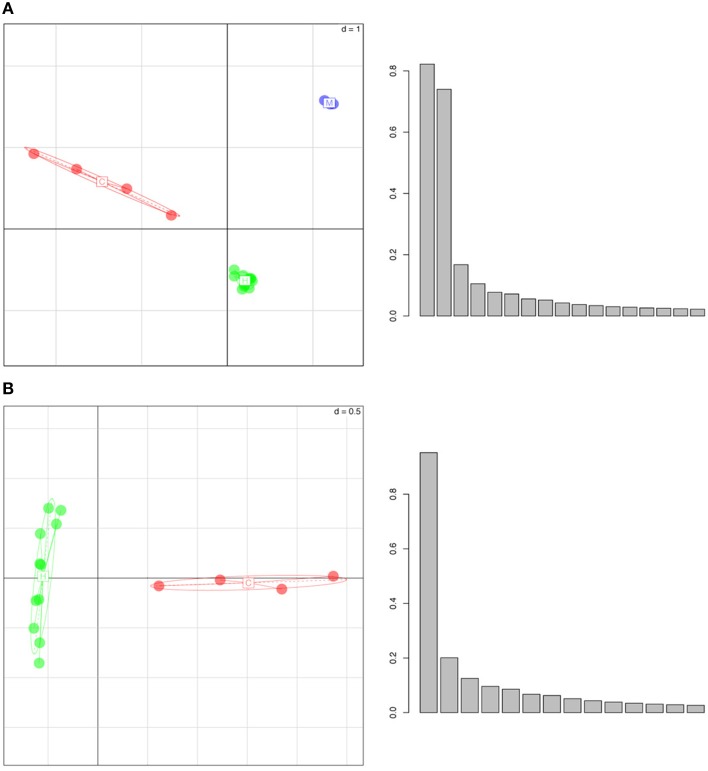
**Whole Genome Principal Component Analysis of peak density values for (A) humans (in green), chimpanzees (in red) and rhesus macaques (in blue), (B) humans (green) and chimpanzees (red)**. The individuals of each species group into well separated clusters, implying that this histone mark has a species-specific signature. In the histograms, y-axes represent the variance (absolute value) and x-axes show the principal components. The first three principal axes represent **(A)** 34.5, 16.3, 13.9 and **(B)** 31.2, 20.5, and 10.5% of the variance, respectively.

Performing a PCA on the whole genome H3K4me3 peak data revealed that individuals of each species cluster together and that different species have distinct, non-overlapping clusters (Figure 1). In the three-species comparison, the first three principal axes describe >60% of the total inertia or total variance of the dataset (34.5, 16.3, 13.9%, respectively). In the two-species comparison (between humans and chimps), the first three principal axes also describe >60% of the total inertia (31.2, 20.5, and 10.5%, respectively). We note that the pair-wise distance based on ellipses of dispersion between humans and chimps is smaller than the distances between humans and macaques or chimps and macaques (Figure 1, Figure S1, and Table S1). Thus, epigenetic marks appear to accumulate differences in a “clock-like” fashion similar to genetic changes, potentially consistent with an important role for genetic drift. However, it is important to note that this observation is based on only a handful of species, and it would be necessary to test across a wider phylogenetic sampling before the assertion could be made strongly.

#### ANEVA

**Figure 2:**
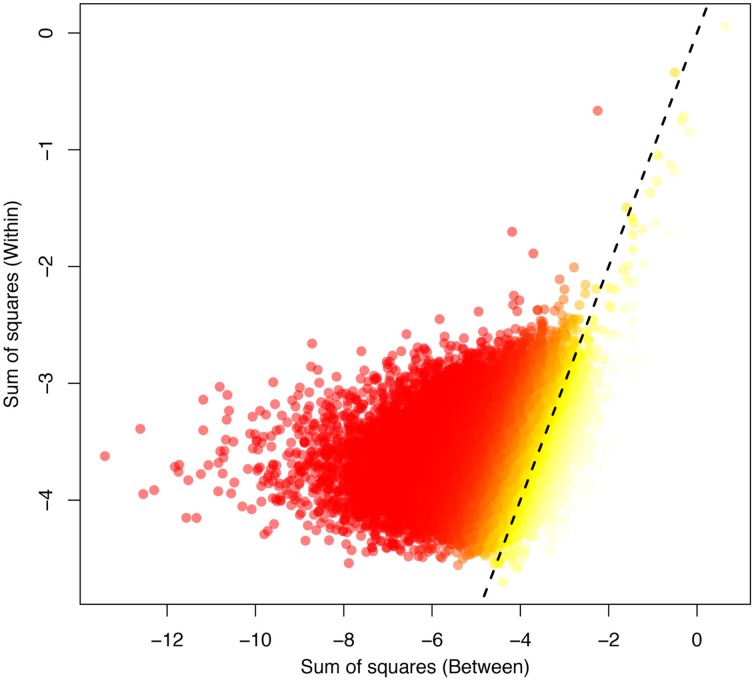
**The between-variation (human-chimp) plotted against the within-variation (human) obtained for the ANEVA**. The highest F_ST_ values are indicated in yellow, corresponding to the most divergent peaks between the species, with low F_ST_ values indicated in red. The dotted line indicates the 1:1 correspondence between the axes.

Over the whole-genome H3K4me3 peak data, epi- F_ST_ was significantly different from zero (*p* < 0.0001, see Figures S2, S4–S6). We plotted the within-SS vs. the between-SS and the epi- F_ST_ values for all peaks for the human and chimp comparison in log scale (Figure 2). We found a weak but significant correlation between *SS_within_* and *SS_between_* (*p* < 2.2e-16 and Spearman's coefficient of correlation of 0.35).

To further investigate the relationship between sequence variation and epigenetic variation, we performed a sliding window analysis (using a window size of 1 Mb and sliding 100 kb at a time) for each chromosome and compared the SNP densities in each window with the average peak density and number of histone peaks per window. We observed weak but significant correlations between the number of SNPs with (1) the number of histone peaks and (2) the average peak density (0.26 and −0.05, respectively; *p* < 2.2e-16). This correlation suggests that the presence of SNPs may indeed have an impact on the enrichment/binding of the histone mark—though it cannot be ruled out that both are being impacted by a third biological correlate.

### Expression analysis

#### Correlating genic variance in H3K4me3 peak densities with differences in gene expression in humans

We identified 404 genes as being differentially expressed in gray matter and 526 genes as being differentially expressed in white matter within humans. Significance of differential expression between any pair of individuals was determined based on *p*-values generated in cuffdiff, specifically when *p* < 0.05. In gray matter, the top 500 genes with the highest variance in H3K4me3 enrichment had a significant overrepresentation of differentially expressed genes (*p* = 0.04). In white matter, no significant overrepresentation was found when considering the top 500 genes with the highest variance (*p* = 0.42). However, the peaks were called from PFC neurons (i.e., gray matter), which may explain the absence of significant results from white matter.

#### Correlating epigenetic F_ST_ with differences in gene expression between humans and chimps

We next evaluated whether our H3K4me3 epigenetic F_ST_ values for genes correlate with differences in gene expression between humans and chimps, using the data for differential gene expression between human and chimps published in Cain et al. (2011). A total of 11,184 genes in this dataset overlapped with the genes for which we calculated epi- F_ST_. In total, 515 are significantly differentially expressed between humans and chimps at the 1% significance level. In addition, 534 of the top 1000 genes identified as having the highest epi- F_ST_ overlapped with this dataset for differential gene expression. Of these, 34 were significantly differentially expressed at the *p* = 0.01 level (FDR corrected *p*-values for differential expression from Cain et al., 2011). We thus find that there is a significant enrichment (*p* = 0.0284) of differentially expressed genes among those with the highest epi- F_ST_. Therefore, epigenetic divergence in H3K4me3 enrichment may explain a fraction of gene expression differences that we observe between species. It is important to note that we review only one histone mark in this study (H3K4me3); in order to capture the full extent of how epigenetic divergence correlates to differences in gene expression between species, it would be helpful to consider several different epigenetic marks. For example, H3K4me3 is the methylation state associated with transcriptional start sites of actively transcribed genes—and further epi- F_ST_ comparisons between transcriptional activation and transcriptional repression marks would be of interest in beginning to quantify differences in pressures. Additionally, it would be of great value in future studies to have paired methylation marks and expression data from identical individuals.

## Conclusions

We developed here a simple model to quantify epigenetic variation—studying the variation in H3K4me3 enrichment using PCA and a newly developed ANOVA-based framework to quantify within- and between-species variation. Differences in H3K4me3 are shown to be correlated with differences in gene expression both within humans and between humans and chimps. Moreover, we observe only a weak correlation between peak density and SNP density. These two results, combined with the increasing evidence for the heritability of epigenetic marks, suggests the potentially important role of epigenetic variation in adaptive evolution. Interestingly, we also found evidence that these marks evolve in a clock-like fashion based on pair-wise distances between species generated from a PCA—though wider species comparisons will be necessary to further evaluate this hypothesis. Thus, these results present an important first step toward quantifying within and between epigenetic variation in the context of a standard population genetic framework, enabling for standardized genomic scan and comparative evaluations of the relative contributions of genetic and epigenetic variation in the adaptive process.

### Conflict of interest statement

The authors declare that the research was conducted in the absence of any commercial or financial relationships that could be construed as a potential conflict of interest.
