# Supplementary material for: Quantifying polymorphism and divergence from epigenetic data: a framework for inferring the action of selection
Source: Front Genet. 2015 May 28;6:190. doi: 10.3389/fgene.2015.00190 (PMC4446996; doi:10.3389/fgene.2015.00190)
Supplement: Supplementary file 1 [file DataSheet1.DOCX]

| **Chromosome** | **Human/Chimp** | **Chimp/Macaque** | **Human/Macaque** |
| --- | --- | --- | --- |
| Chr 1 | 0.02124189 | 0.04891031 | 0.05116148 |
| Chr 2 | 0.02146954 | 0.04369563 | 0.04753389 |
| Chr 3 | 0.01737492 | 0.04259818 | 0.04678319 |
| Chr 4 | 0.01756254 | 0.04183299 | 0.04473479 |
| Chr 5 | 0.0196392 | 0.04245182 | 0.04743993 |
| Chr 6 | 0.01770319 | 0.04491983 | 0.04646567 |
| Chr 7 | 0.02099689 | 0.04944921 | 0.05644539 |
| Chr 8 | 0.01856389 | 0.04333441 | 0.04454668 |
| Chr 9 | 0.02310557 | 0.04803338 | 0.05202952 |
| Chr 10 | 0.01956363 | 0.04392136 | 0.04571862 |
| Chr 11 | 0.02176953 | 0.04600331 | 0.04866042 |
| Chr 12 | 0.02158077 | 0.05137097 | 0.05269849 |
| Chr 13 | 0.0187142 | 0.04373666 | 0.04812125 |
| Chr 14 | 0.01851303 | 0.05046364 | 0.05040242 |
| Chr 15 | 0.02104753 | 0.04562709 | 0.04815239 |
| Chr 16 | 0.02714027 | 0.05578123 | 0.05991553 |
| Chr 17 | 0.02848009 | 0.06524152 | 0.06626173 |
| Chr 18 | 0.01819085 | 0.04110929 | 0.03869237 |
| Chr 19 | 0.04482121 | 0.0789726 | 0.08712528 |
| Chr 20 | 0.02259333 | 0.04502349 | 0.05199839 |
| Chr 21 | 0.02685079 | 0.05014051 | 0.05754766 |
| Chr 22 | 0.02838024 | 0.04853782 | 0.05926149 |
| Chr X | 0.01087214 | 0.03794095 | 0.03650992 |

Supp Table 1. Average Euclidean distances between the centers of ellipses of dispersion obtained by performing a PCA on the peak densities for each chromosome.

Supp Fig1. Average genome-wide H3K4me3 peak density distributions for humans (green), chimps (red) and macaques (blue) in prefrontal cortex neurons. Humans and chimps have more similar distributions compared to macaques.

Supp Figure 2. Distribution of genome wide average between- and within-sum of squares obtained from an ANOVA for the three species using 10000 permutations to assign different individuals randomly to one of the three species (humans, chimps, or macaques). The maximum between-species variation and the minimum within-species variation is observed when the individuals are correctly assigned to their respective species (shown by the red line on the two graphs).

Supp Figure 3. Comparison of the p-values of the likelihood ratio test of dN/dS=1 versus dN/dS>1 (Nielsen et al. 2005) and the epi-F_ST_ values for humans and chimps. No significant correlation is observed.

Supp Figure 4.: Genome-wide distribution of Euclidean distances between the centers of the ellipses of dispersion (from PCA) for each pair of species: humans/chimps (red), chimps/macaques (blue), and humans/macaques (green).

Supp Figure 5: Distribution of Genome wide epi- F_ST_ values, with the median and mean values indicated in red and blue, respectively.

Supp Figure 6: Density plot of epi- F_ST_ values per chromosome.
